# Do health policy advisors know what the public wants? An empirical comparison of how health policy advisors assess public preferences regarding smoke-free air, and what the public actually prefers

**DOI:** 10.1186/2045-4015-2-20

**Published:** 2013-05-21

**Authors:** Laura J Rosen, David A Rier, Greg Connolly, Anat Oren, Carla Landau, Robert Schwartz

**Affiliations:** 1 Department of Health Promotion, School of Public Health, Sackler Faculty of Medicine, Tel Aviv University, POB 39040, Ramat Aviv, 69978, Israel; 2 Department of Sociology and Anthropology, Bar-Ilan University, Ramat-Gan, 52900, Israel; 3 Division of Public Health Practice Department of Society, Human Development, and Health Harvard School of Public Health, Harvard University, Landmark Building, 677 Huntington Avenue, Boston, MA, 02115, USA; 4 Cohen Institute, B.I. Cohen Institute for Public Opinion Research Tel Aviv University, POB 39040, Ramat Aviv, 69978, Israel; 5 Ontario Tobacco Research Unit, Dalla Lana School of Public Health, University of Toronto, 155 College Street, Toronto, Ontario, M5T 3M7, Canada

**Keywords:** Health policy, Public opinion: Secondhand smoke exposure (SHS, SHSe), Tobacco smoke exposure, Smoke-free, Tobacco control

## Abstract

**Background:**

Health policy-making, a complex, multi-factorial process, requires balancing conflicting values. A salient issue is public support for policies; however, one reason for limited impact of public opinion may be misperceptions of policy makers regarding public opinion. For example, empirical research is scarce on perceptions of policy makers regarding public opinion on smoke-free public spaces.

**Methods:**

Public desire for smoke-free air was compared with health policy advisor (HPA) perception of these desires. Two representative studies were conducted: one with the public (N = 505), and the other with a representative sample of members of Israel’s health-targeting initiative, Healthy Israel 2020 (N = 34), in December 2010. Corresponding questions regarding desire for smoke-free areas were asked. Possible smoke-free areas included: 100% smoke-free bars and pubs; entrances to health facilities; railway platforms; cars with children; college campuses; outdoor areas (e.g., pools and beaches); and common areas of multi-dweller apartment buildings. A 1–7 Likert scale was used for each measure, and responses were averaged into a single primary outcome, DESIRE. Our primary endpoint was the comparison between public preferences and HPA assessment of those preferences. In a secondary analysis, we compared personal preferences of the public with personal preferences of the HPAs for smoke-free air.

**Results:**

HPAs underestimated public desire for smoke-free air (Public: Mean: 5.06, 95% CI:[4.94, 5.17]; HPA: Mean: 4.06, 95% CI:[3.61, 4.52]: p < .0001). Differences at the p = .05 level were found between HPA assessment and public preference for the following areas: 100% smoke-free bars and pubs; entrances to healthcare facilities; train platforms; cars carrying children; and common areas of multi-dweller apartment buildings. In our secondary comparison, HPAs more strongly preferred smoke-free areas than did the public (p < .0001).

**Conclusions:**

Health policy advisors underestimate public desire for smoke-free air. Better grasp of public opinion by policy makers may lead to stronger legislation. Monitoring policy-maker assessment of public opinion may shed light on incongruities between policy making and public opinion. Further, awareness of policy-maker misperceptions may encourage policy-makers to demand more accurate information before making policy.

## Background

Health policy-making, and the implementation of approved policies, is a complex, multi-factorial process [1]. The policy-making process requires balancing conflicting values. These include: the role of government in protecting the health of individuals or the public; the rights of individuals to choose to pursue unhealthy lifestyles; and corporate freedom to promote and market products--even if they may be unhealthy for specific individuals or for populations. Such inherent conflicts are present regarding an array of products related to health, including high-sugar, high-fat, and high-sodium foods, alcohol, drugs, and tobacco.

The process is complicated by financial considerations, as governments, ostensibly the protectors of citizens, may directly or indirectly benefit from the marketing of such products. Both types of benefits are salient in the case of tobacco. In most countries, indirect benefits such as tax revenues accrue from sale of tobacco products. The proportion of income from tobacco is substantial in many countries: according to the WHO, governments annually collect more than US $167 billion in tobacco tax revenues [2] (p. 62). Benefits may also be direct: the government of Japan, for example, is the major shareholder in Japan Tobacco and owner of roughly half of its market shares [3]. Thus, Japan has a clear conflict of interest regarding tobacco control and the finances of the country. Over 7% of China’s total governmental revenue comes from tobacco [4] (p. 56). Beyond this, multi-national tobacco companies today seek to limit the autonomy of nations in making tobacco control policy, in efforts to maintain their corporate profits [5].

A further issue for health policy-makers and implementers of such policies is public support for policies. When public support is high, laws may be more likely to be passed and implemented [6]. On the other hand, when public support is low, policy-makers may be loath to pass or enforce measures, and implementers may subsequently be unwilling to enforce them. A recent qualitative study from Israel described police reluctance to enforce smoke-free air policy, due, in part, to perceived unpopularity of the law among bar and pub owners and their patrons [7].

Though public opinion has some influence on policy, the association between public support and policy is far from perfect. Beyond the complexity of the policy-making process, along with a host of competing interests and demands, an additional reason for this limited impact of public opinion may be that policy makers hold misperceptions of actual public opinion. In researching the field of US foreign policy, Kull and Ramsey [8] stated: “much research has shown that policy decisions can be greatly influenced by misperceptions, just as much as by objective factors.” They identified two main contributors to policymaker misperceptions about public opinions: failure on the part of policymakers to try to understand public opinion, and “a tendency to assume that the vocal public is representative of the general public.” (p. 115) Moreover, they found that policymakers often dismissed the validity of data from public opinion polls.

### Smoke-free areas: a prototype for conflict in health policy-making

At the policy level, the question of desirability of smoke-free areas involves natural conflicts between different stakeholders. First, smokers are generally less supportive of restrictions on smoking in public places than are non-smokers [9]. Second, governments, the health care system, and non-smokers are concerned that exposure to secondhand smoke causes premature death and disease among nonsmokers [10]. Third, local and multinational tobacco companies have a vested interest in keeping tobacco control actions to a minimum [5]. Fourth, despite evidence to the contrary [2], p. 31, hospitality venues often perceive that restrictions on smoking are detrimental to affected businesses.

In this health-related area, research on perceptions of policy makers regarding public opinion is scarce. In one qualitative study of policy-maker opinions regarding smoke-free cars with child passengers in New Zealand, Thomson et al. [11] (p. 970) found that the policy community lacked awareness “of national-level public support for banning smoking in cars with children.” That study was based on qualitative interviews with policy makers, not on direct comparisons between policy maker perceptions of public opinions and actual public opinions, and was not generalizable. We are unaware of any past research which quantitatively measures the accuracy of policy-maker assessment of public opinion in the area of tobacco control.

The present study attempts to help fill this gap in the literature, as it compares public desire for smoke-free air with decision-maker perception of that public desire. It is based on two representative studies, one with the public, and the other with health policy advisors. The two studies were run during the same time period, and included corresponding questions regarding desire for smoke free areas. For each question about the desire of the respondents participating in the public survey about smoke-free air, there was a question for the health policy advisors about their opinion about public attitudes towards smoke-free areas. The study was conducted at a time when a national tobacco control plan, which included new regulations for smoke-free air, was being written. The findings are discussed in the context of the policy-making effort in progress at the time of the study, and regulations subsequently approved by the government and passed by the Israeli *Knesset* (Parliament).

## Methods

### Overview

We conducted two surveys regarding public preferences for smoke-free air in December, 2010 [12]. One was a representative survey of Israeli adults (N = 505, response rate = 61%) which assessed, among other topics, the Israeli public’s support for smoke-free environments. The second was based on a random sample (N = 34, response rate = 64%) of Israeli civil servants, professionals and stakeholders who were members of Healthy Israel 2020, Israel’s Ministry of Health-sponsored health targeting initiative. We describe the sampling aspects of each survey, the response and explanatory variables, and specify statistical methods which we used to compare Israeli public opinion with policy-maker assessment of those opinions.

### Sampling strategy and conduct: public opinion

Between November 30th, 2010 and December 22nd, 2010, a national phone survey of Israeli adults was conducted by the B. I. and Lucille Cohen Institute for Public Opinion Research of Tel-Aviv University. Interviews were conducted in Hebrew, Arabic, or Russian, according to the preference of the interviewee. The survey was representative of adults aged 18 and above living in private residences with land telephone lines. The study was approved by the Tel Aviv University Ethics Committee.

In all, 1077 phone numbers were selected for inclusion. Of these, 168 (16%) numbers proved to be disconnected throughout the entire survey period; 47 (4%) were faxes or modems; and 33 (3%) were businesses. Of the remaining 829 (77%) phone numbers available for the study, full interviews were obtained from 505, giving a response rate of 61%. Of the 324 non-respondents, there were 188 (23%) refusals, 84 (10%) numbers which didn’t answer, 44 (5%) were excluded due to communication problems (individuals didn’t speak Hebrew, Russian, or Arabic, or had difficulties in hearing or understanding), and 8 (1%) were partial interviews. The final sample included 505 participants, of whom 424 (83.9%) were from the Jewish sector and 81 (16.1%) were from the Arab sector. Full details of the methodology, as well as selected results, have been published previously [9].

### Sampling strategy and conduct: health policy-advisors (“HPAs”)

We used the master list of appointees to the Israeli health-targeting initiative, Healthy Israel 2020, as our sampling frame. Healthy Israel was spearheaded by the Israeli Ministry of Health in 2005 [13]. The goals of the initiative were to set health targets and identify evidence-based strategies to achieve them. Several hundred professionals were appointed to the original 21 committees. Included in the effort were a broad range of civil servants, stakeholders and professionals hailing from the government (Health Ministry, Education Ministry, Finance Ministry), academia, all four of Israel’s Health Maintenance Organizations, and non-governmental organizations. Physicians, nurses, epidemiologists, statisticians, educators, communications experts, policy analysts and economists were represented. Because the role of Healthy Israel 2020 members was to advise the Ministry of Health regarding health policy, we term this group “Health Policy Advisors (“HPAs”).

Sampling for the survey was conducted in August, 2010. Of the 265 members on the 2020 Master List (dating from October, 2006), 14 members were excluded because they had previously taken part in a related (but independent) qualitative survey of policy-maker opinions [14]. Sixty names were randomly sampled from the list of the remaining 251 names. Of the 60 individuals sampled, six of the individuals were not in the country at the time of the survey and were excluded from the study, leaving a final sample of 54 HPAs.

The survey was conducted in December, 2010.

### Questionnaire

Questions regarding desire for smoke-free places were informed by a previous survey conducted in Russia [15]. An early version of the questionnaire was validated using a test-retest approach, with an interval of one week, on a population of 20 individuals. In addition, all questions were piloted on a separate sample of 15 individuals. Most of the questions were originally written in English or taken from English publications. The questionnaire was professionally translated into Hebrew, Arabic, and Russian.

We asked about desire for smoke-free areas. Eight locales were included: 100% of bars and pubs; entrances to healthcare facilities; train platforms; cars carrying children; open areas of college campuses; open outdoor areas such as beaches, parks, and swimming pools; and common areas of multi-dweller apartment buildings; and school buildings. The question asked of the public for the first seven places was:

*To what extent in your opinion should the following places be, or not be, totally smoke free places? Please rate your answers on a 1–7 scale where 1* = *The place doesn’t have to be smokefree and 7* = *The place must be smokefree*

The corresponding question asked of the policy-makers was

*To what extent in your opinion, does the Israeli public want or not want that the following places be, or not be, totally smoke free places? Please rate your answers on a 1–7 scale where 1* = *The place doesn’t have to be smokefree and 7* = *The place must be smokefree.*

The question of the public regarding school buildings was:

The following questions are about school staff. In your opinion, should schools in Israel be: 1 – without staff smoking rooms, or 2- with staff smoking rooms?

The question for the policy makers was:

The following question concerns school staff. In your opinion, does the Israeli public want 1- schools without staff smoking rooms, or 2- schools with staff smoking rooms?

### Scales

We built a scale (“Desire”) for support for smoke-free places by averaging the seven questions asked on the 1–7 point Likert scale (100% of bars and pubs; entrances to healthcare facilities; train platforms; cars carrying children; open areas of college campuses; open outdoor areas such as beaches, parks, and swimming pools; and common areas of multi-dweller apartment buildings).

We examined the desire for 100% smoke-free schools, i.e., those without staff smoking rooms, on a binary (yes/no) basis.

### Statistical analyses

We performed three sets of analyses. The first set concerned the primary comparison, between public preferences for smoke-free places and HPA assessment of that opinion. The major endpoint in that analysis was DESIRE, and secondary endpoints were defined as the seven individual places of interest which were measured on the Likert scale. These analyses were done using t-tests, a type of parametric analysis, which is justified if a scale has 7 points [16].

Since the smoke-free schools variable, measured as a binary response, was excluded from DESIRE, we used instead a Chi-squared test to compare public desire with HPA assessment of that desire.

The second set of analyses was based on the secondary comparison (public preferences for smoke-free areas with HPA preferences for smoke-free areas), with Desire as the endpoint. As before, we employed a t-test.

The third set of analyses performed involved exploration of correlates of HPA assessment (Desire). This was done using multiple analysis of variance (PROC GLM). Fixed categorical variables were sex (male/female), smoker (current, former, never), age category (<=49, 50–59, 60+), family financial status (medium, high/very high), and exposure to secondhand smoke in past week (at least 4×/week, 2-3×/week, 1×/week or less).

All analyses were done using SAS Version 9.2.

## Results

Table 1 presents socio-demographic information, and information about smoking status, for the sample of the Israeli public and of the HPAs. Participants in the HPA sample were older than were participants in the public sample (HPA-Mean: 57.4, Std: 9.8, Public-Mean: 46.9, STD: 16.9, p < .0001), better educated (p < .0001), more likely to be Jewish (HPAs: Jews: 96.1%, Public: Jews: 83.1%, p = .0315), and had higher family financial status (p < .0001). Smoking status differed between the two samples (p = .0380). While in both groups about half were never smokers (HPAs: 55.9%, Public: 50.8%), in the HPA group there were no current daily smokers, as opposed to 17.9% among the public. The HPAs included more former smokers (HPAs: 35.3%, Public: 26.9%).

**Table 1:** Demographic distributions of respondents from public and policy maker surveys (Dec. 2010)

**Variable**	**Category**	**Health policy advisor**	**Public**
Sex (P **=** .6816)	Male	52.9%	49.3%
	Female	47.1%	50.7%
Religion (p **=** .0315)	Jewish	96.1%	83.1%
	Other (Muslim, Druze, Christian, not specified)	2.9%	16.9%
Education ( P < .0001)	Up to 12	0.0%	47.7%
	Matriculation Certificate	0.0%	6.0%
	College/Seminary	0.0%	13.6%
	Academic	100%	32.7%
Family Financial Status (P < .0001)	Very high	3.0%	4.1%
	High	69.7%	17.6%
	Medium	27.3%	62.4%
	Low	0.0%	12.1%
	Very low	0.0%	3.9%
Smoking Status (P **=** .0380)	Daily smoker	0%	17.9%
	Occasional smoker	8.8%	4.4%
	Former smoker	35.3%	26.9%
	Never smoker	55.9%	50.8%

As Table 2 indicates, reported monthly exposure to secondhand smoke was about 80% in both samples. None of the HPAs, but about a third of the public, were exposed to secondhand smoke at home (p < .0001). Exposures at work, hospitality venues, and public transportation were higher among the public than among the HPAs (Workplace--HPAs: 26.5%, Public: 45.6%; Hospitality--HPAs: 29.4%, Public: 43.3%; public transportation--HPAs: 29.4%, public: 35.4%). However, these differences did not reach statistical significance. Nearly 40% of both groups were exposed elsewhere; on examination of individual responses, this often referred to events such as weddings. Participants in the public sample reported more frequent exposure (p = .0059), with 33.9% reporting daily exposure, as opposed to 5.9% daily exposure among HPA sample participants. Participants in the public sample also reported greater cumulative hours of weekly exposure, with 17.1% of the public sample, versus only 3.0% of the HPA sample, reporting 2–8 hours of weekly exposure (p = .0067).

**Table 2:** Exposure to secondhand smoke

**Variable**		**Health policy advisor**	**Public**	**p-value**
**Exposure during past month, in various places**	Anyplace% Exposed	82.4	80.2	.7596
	Home% Exposed	0	32.1	<.0001
	Work% Exposed	26.5	45.6	.0303
	Café, bar, or pub% Exposed	29.4	43.3	.1140
	Public transportation% Exposed	29.4	35.4	.4811
	Elsewhere% Exposed	39.4	39.0	.9598
**Frequency of exposure during regular week**				
	Daily	5.9	33.9	.0059
	4-6 times/week	2.9	6.2	
	2-3 times/week	26.5	13.4	
	Once per week	23.5	15.8	
	Never	41.2	30.7	
**Hours of exposure during past week**	>8	0	13.9	.0067
	2-8	3.0	17.1	
	0-2	57.6	37.4	
	None	39.4	31.6	

### Comparisons regarding smoke-free areas

Table 3 presents descriptive information for desire for smoke-free places as an average of a 1–7 scale, for each one of the seven places individually and for the combined measure, for: public opinion; HPA opinion; and HPA assessment of public opinion.

**Table 3:** Comparison of support for smoke-free spaces: public opinion, health policy maker opinion, and health policy advisor assessment of public opinion

	**Group**								
	**A. Public opinion**			**B. Health policy advisor opinion**			**C. Health policy advisor assessment of public opinion**		
	**Mean**	**Std**	**N**	**Mean**	**Std**	**N**	**Mean**	**Std**	**N**
Bars and pubs 100% (p = .0069) ^1^	5.21	2.35	473	6.35	1.23	34	4.29	1.70	31
Health care entrances (p < .0001) ^1^	6.53	1.41	501	6.71	0.72	34	4.73	1.86	30
Train platforms (p = .0170) ^1^	4.59	2.51	498	5.82	1.36	33	3.68	1.94	31
Cars with children (p = .0021) ^1^	6.63	1.27	503	6.91	0.51	34	5.48	1.88	31
College campus (p = .4573) ^1^	3.44	2.52	499	4.44	1.88	34	3.10	2.02	31
Parks, beaches, pools (p = .3371) ^1^	3.00	2.38	498	4.15	1.71	34	2.58	1.96	31
Multi-dweller apt. common areas (p = .0001)^1^	5.95	1.92	500	6.50	1.02	34	4.58	2.08	31
Desire (constructed variable) ^1^ (p < .0001)	5.06	1.33	504	5.84	0.75	34	4.06	1.24	31

^1^ Denotes comparison A. Public Opinion vs. C. Health Policy Advisor assessment of public opinion.

For all measures including the combined measure, there was positive and fairly strong support for stronger policies. HPA assessment of public opinion was lowest, actual public opinion was in the middle, and HPA’s own opinion was the highest.

#### Analysis – set 1: comparisons between public opinion and HPA assessment of public opinion

Comparisons of public desire for SHS and HPA assessment of that opinion showed that HPAs underestimated public desire for smoke-free areas on every measure. For the primary endpoint DESIRE, public support was a full point higher, on the 1–7 scale, than HPAs assumed it to be (Public: Mean: 5.06, [4.94, 5.17]: HPA: Mean: 4.06 [3.61, 4.52]: p < .0001). These differences were statistically significant for the overall measure (p < .0001), and were less than .05 for five of the seven measures: 100% smoke-free bars and pubs; entrances to healthcare facilities; train platforms; cars carrying children; and common areas of multi-dweller apartment buildings.

Smoke-free schools (i.e., schools without staff smoking rooms) were preferred by 47.6% of the public; only 36.7% of the HPAs believed that the public preferred smoke-free schools. This difference was not statistically significant (p > .05).

#### Analysis set 2: comparisons between public opinion and HPA opinion

This secondary analysis compared the combined outcome variable DESIRE for smoke-free spaces between the public and the HPAs. HPAs expressed significantly higher levels of desire for smoke-free areas than did the public (p < .0001).

**Figure 1:**
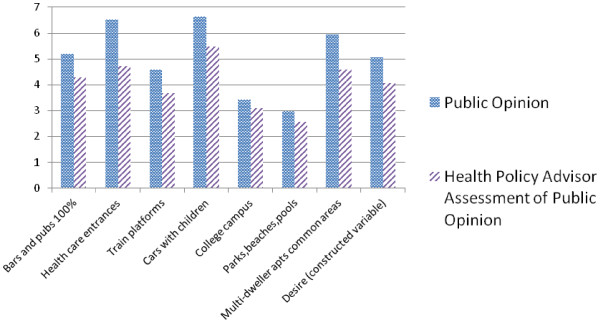
Public opinion, and health policy advisor assessment of public opinion, regarding desire for smoke-free air.

Figure 1 presents, for our primary comparison of public opinion vs. HPA assessment of that opinion, descriptive information on the 8 variables: the seven which were asked on the 1–7 Likert scale, plus the scaled summary variable, “DESIRE.”

#### Analysis set 3: correlates of HPA assessment

Thirty observations were included in this exploratory multivariate analysis of covariance, with the response variable defined as HPA assessment of public desire. Family financial status was the only variable which reached statistical significance (p = .0038). Mean HPA assessment was higher in the medium income group than in the high/very high income group (LSMeans: High Income Group: 3.51; Medium Income Group: 5.06). The R^2^ for the model was .57.

## Discussion

In this study, we compared public opinions with how members of the health policy community perceived those opinions during the same time period. The primary finding was that health policy advisors (HPAs) consistently underestimated the public’s desire for smoke-free air. This finding was statistically significant for our main comparison, a variable constructed from seven independent places. Five of the seven statistical tests showed p-values of less than .05, and directionality was maintained across all seven individual components. The pattern of support and perceived support was similar for all variables.

Our data cannot pinpoint precisely why HPAs underestimated public support. To that end, future studies, perhaps using qualitative methods, might examine questions such as *meaning*: when the public and the HPAs consider stronger regulation, how do they, respectively, view it? How do they relate to contemporary controversies over issues such as the “nanny state”? What is it about their own experiences that shapes their views? Moreover, what other factors influence attitudes in the different sectors? Media coverage might be an especially fruitful area for future research. For example, some media coverage and blog posts feature “embattled smokers” exiled from their homes and offices, forced to smoke outside or in cramped, inconvenient “smoking areas”. Lacking empirical data on actual public attitudes, HPAs--assuming that such coverage reflects, or even shapes, public opinion--might use media coverage as a proxy for public opinion on smokefree regulation (in fact, perhaps public support would be even higher without such coverage). This might help explain why HPAs underestimated public support.

The study also found that HPAs’ own desire for smoke-free places was significantly stronger than that of the public, and that HPA personal desire for smoke-free places was consistently higher than was their assessment of the public’s desire. The exploratory analysis suggested that HPAs with high or very high family incomes underestimated the public desire for smoke-free air to a greater degree than did middle-income HPAs. Our present data are insufficient to explain this last finding. However, we note that HPAs with the highest incomes are (nearly by definition) likeliest to be remote—spatially and culturally--in terms of employment, residence, mode of transportation, sources of information, education, leisure activities, etc., from the lives of the “average” citizen. This comparative isolation—particularly when accurate, current empirical data on actual public attitudes are unavailable—may hamper these HPAs’ ability accurately to gauge public sentiment. Future studies might look more carefully at this question.

### Strengths, limitations, and generalizability

The major strength of this research is that it compared, using corresponding questions, public attitudes towards smoke-free areas, and HPA perceptions of those attitudes. Both studies were representative, and the studies were performed simultaneously.

This study has three limitations. First, the small sample size of the HPA survey precludes in-depth or even definitive analyses of correlates of HPA perceptions. Yet, the analysis suggests directions for further study which could contribute to deeper understanding of HPA opinions. Second, our HPA category included those involved in health policy-making in the specific context of Healthy Israel 2020 [13]. Other individuals who are involved in the creation of health policy, outside of this context, are not represented here. Third, the public survey was based on a sampling of landlines. This may, in principle, have led to under-sampling of subpopulations likely to use cell phones exclusively or of very poor people. However, the sampling strategy produced distributions of measured variables (nationality, gender, and education level) which were almost identical to population-wide distributions [9].

Finally, our findings should, broadly speaking, illuminate the public-policymaker dynamic also for other health issues. However, that dynamic might well play out somewhat differently across these different issues. Factors such as the overall public salience of the issue, the extent of media coverage, and the degree of scientific consensus are likely to be especially relevant. One question about generalizing the present data involves the scientific basis for anti-tobacco regulation. Smoking is unusual in there being so wide a scientific consensus, and public awareness, about its danger. Even if consensus and awareness regarding, specifically, second-hand smoke are weaker, smoking is a more clear-cut risk, in the eyes of the public, than are many other types of risks. It remains to be demonstrated empirically to what extent the public favor regulation in areas such as nutrition, where the scientific consensus is weaker and more liable to change or even reversal on the health implications of carbohydrates, dietary fats, etc.

### Further thoughts: public opinion and policy--commonly accepted wisdom

The relationship between public opinion and policy has been studied for decades. In cases where an association has been found, the central questions concern direction of effect: Does public opinion affect policy? Does policy affect public opinion? Might there be some common factor which affects both, simultaneously? Or, is this a complex system characterized by a “feedback loop” in which they affect each other? It has been hypothesized that information on public opinion before and after changes in policy may provide some insight into directionality [17].

In the case of secondhand smoke laws, each of these approaches has proponents. The importance of public opinion on policy was stressed by the WHO’s International Agency for Research on Cancer, which wrote that, “In democratic nations, supportive public attitudes are often necessary for facilitating the process of passing smokefree legislation or regulations by local or national governments” [6] (p. 93). Recently, however, some other researchers have concluded that the reverse direction is correct, and that policy affects public opinion: on the basis of data collected before and after policy changes regarding smoke-free laws, they concluded that: “although the initial debate over smoke-free policies may be tumultuous, once people understand the rationale for implementing smoke-free policies and experience their benefits, public support increases even among smokers [18] (p. 642).” The third scenario – that some other factor is simultaneously affecting both policy and public opinion – is supported by recent research examining the importance of scientific information on policy: researchers suggest that the emergence of scientific evidence documenting the harm from secondhand smoke propelled the adoption of smoke-free laws in many countries [19]. While the researchers restrict their generalizations to the creation of policy, and don’t specifically mention public opinion, it could be that the increasingly compelling science base for the harms of secondhand smoke exposure may have affected both public opinion and policy makers, thus advancing the policy-making agenda. Finally, it has been suggested that there exists a “feedback loop” whereby public support enables stronger policy, which in turn leads to even higher levels of public support (perhaps due to the public education around the adoption and implementation of stronger policy) [20]. Feedback loops are a characteristic of complex adaptive systems. It is also possible that each of these four approaches may play a role in the total picture.

### Findings in the context of Israeli policy-making

Both of the surveys described in this paper were conducted in December, 2010, at the same time as the Israel Public Committee for Reducing Harm Due to Smoking, convened by the Deputy Minister of Health, was finalizing its recommendations for a national tobacco control plan [21]. As a basis for that plan, the Committee used the recommendations made by the Healthy Israel 2020 Tobacco Control Subcommittee [22]. Many, but not all, of the original 2020 recommendations were adopted by the Public Committee. The 2020 recommendations included limited recommendations for regulation of outdoor public spaces (“smoking in special open spaces like public swimming pools, beaches, bus stops and train stations”) [22] (Supplementary Table Two, http://www.health-policy-systems.com/content/8/1/17/additional). Additional recommendations, including a ban on smoking at entrances to healthcare facilities, were adopted by the Public Committee. However, other possible recommendations were considered but rejected. One of the 2020 recommendations not adopted by the Public Committee was the call for a ban on smoking in cars carrying children. That recommendation was not adopted because of concerns that the Israeli public would not accept intrusion into the “private spaces” of individual cars. Perhaps, had the data on public desire for smoke-free cars carrying children (general population: 94% support, with even 90% of *smokers* agreeing) been available, the final outcome may have differed.

The Israeli Cabinet approved the recommendations of the Public Committee for Reducing Harm Due to Smoking in May, 2011 [21]. Many of the elements of that plan required full *Knesset* (parliamentary) approval, and some elements were approved in May, 2012 and implemented in July 2012. Table 4 compares Healthy Israel 2020 recommendations on these items, recommendations by the Public Committee which were approved by the Cabinet in 2011, and current Israeli law.

**Table 4:** Comparisons of recommendations by healthy Israel 2020, the Public Committee for Reducing Harm Due to Smoking, and current Israeli law on 8 items (as of March 2013)

**Regulation**	**Healthy Israel 2020**	**Public Committee**	**Current Israeli law**
**100% Smoke-Free Bars and Pubs**	Yes (not specifically mentioned, but subsumed under recommendation for 100% smoke-free workplaces)	Partial. To existing law, added 75% of outdoor areas of bars and pubs	Partial restrictions 75% of outdoor areas of bars and pubs smoke-free (direct result of Public Committee recommendation)
**Entrances to healthcare facilities**	No	Yes	Restricted (direct result of Public Committee recommendation)
**Train platforms**	Yes	Yes	Restricted (direct result of Public Committee recommendation)
**Cars carrying children**	Yes	No	No restrictions
**Open areas of college campuses**	No	No	No restrictions
**Outdoor open areas such as parks, beaches, and swimming pools**	Yes	Swimming pools	No smoking in swimming pools (direct result of Public Committee)
**Common areas of multi-dweller apartment buildings**	No	No	No restrictions
**Smoke-free schools (no staff smoking rooms)**	Yes (not specifically mentioned, but subsumed under recommendation for 100% smoke-free workplaces)	No	No

## Conclusions

Health policy advisors (HPAs) underestimate public desire for smoke-free air. Inaccurate understanding of public opinion by policy makers may have important ramifications, including weaker tobacco control legislation than the public is actually ready to accept. Monitoring policy-maker assessment of public opinion, and adopting it as a mediating variable on policy, may shed light on incongruities between policy making and public opinion. Further, drawing attention to policy-maker misperceptions may encourage them to demand more accurate information before making policy in future. In the present case, for example, this might involve regular national surveys of public attitudes and support regarding smoke-free areas and regulation of second-hand smoke exposure in various domains. Indeed, the present study indicates a public mandate for change in this regard.

More generally, however, it is only fitting that policy analysts in a democratic society have accurate data on the wishes of the public on whose behalf they work.

## Abbreviations

HPA: Health Policy Advisor; WHO: World Health Organization.

## Competing interests

The authors declared that they have no competing interest.

## Authors’ contributions

LJR conceived the idea of the study, directed both surveys, performed all statistical analyses, and wrote the initial draft of the paper. DAR contributed to the design of the study, to the writing, and to data interpretation. RS provided the idea for the initial public opinion survey and contributed to the writing. GC contributed to the development of the research, to the content of both questionnaires and to interpretation of the data. AO contributed to the design of both questionnaires, and ran the public survey. CL assisted with the questionnaire for the policy makers and was the interviewer for the Health Policy Advisor survey. All authors reviewed drafts of the manuscript and gave final approval for publication.

## Authors’ information

LJR is Chair of the Health Promotion Dept. at the School of Public Health at Tel Aviv University. She is former National Coordinator of Healthy Israel 2020, Committee Head of the Tobacco Control Subcommittee of Healthy Israel 2020, and a member of the Public Committee for Reducing Harm due to Smoking in Israel. Her research focuses on tobacco use and exposure, and evidence-based public health.

DAR is Senior Lecturer in the Department of Sociology & Anthropology at Bar-Ilan University, and adjunct faculty member in the Hebrew University-Hadassah Braun School of Public Health and Community Medicine.

GC is Professor of the Practice of Public Health and Director, Center for Global Tobacco Control at the Harvard School of Public Health.

AO is with the Cohen Institute for Policy Opinion Research at Tel Aviv University.

CL is an MPH student in the Health Promotion Track at the School of Public Health at Tel Aviv University.

RS is Associate Professor at the Dalla Lana School of Public Health and Executive Director and Principal Investigator, Ontario Tobacco Research Unit, University of Toronto.
